# Comparative Physiological and Proteomic Analysis Reveals Different Involvement of Proteins during Artificial Aging of Siberian Wildrye Seeds

**DOI:** 10.3390/plants9101370

**Published:** 2020-10-15

**Authors:** Xiong Lei, Wenhui Liu, Junming Zhao, Minghong You, Chaohui Xiong, Yi Xiong, Yanli Xiong, Qingqing Yu, Shiqie Bai, Xiao Ma

**Affiliations:** 1 College of Animal science and Technology, Sichuan Agricultural University, Chengdu 611130, China; lxforage@126.com (X.L.); Junmingzhao163@163.com (J.Z.); xiongyi95@126.com (Y.X.); yanlimaster@126.com (Y.X.); yuqinggzu93@126.com (Q.Y.); 2 Sichuan Academy of Grassland Science, Chengdu 611731, China; ymhturf@163.com; 3 Key Laboratory of Superior Forage Germplasm in the Qinghai-Tibetan Plateau / Qinghai Academy of Animal Science and Veterinary Medicine, Xining 810016, Qinghai, China; qhliuwenhui@163.com; 4 College of Environmental Sciences, Sichuan Agricultural University, Chengdu 611130, China; sophyx1@163.com

**Keywords:** *Elymus sibiricus*, seed aging, isobaric tandem mass tag labeling, reactive oxygen species, parallel reaction monitoring

## Abstract

Seed aging has an important effect on the germplasm preservation and industrialized production of Siberian wildrye (*Elymus sibiricus*) in the Qinghai-Tibet Plateau. However, so far its underlying molecular mechanisms still largely remain unknown. To shed light on this topic, one-year stored seeds of *E. sibiricus* were exposed to artificial aging treatments (AAT), followed by seed vigor characteristics and physiological status monitoring. Then global proteomics analysis was undertaken by the tandem mass tags (TMT) technique, and the proteins were quantified with liquid chromatography-tandem mass spectrometry on three aging time points (0 h, 36 h and 72 h). Finally, we verified the expression of related proteins by parallel reaction monitoring (PRM). Our results demonstrated that the seed vigor decreased remarkably in response to artificial aging, but the relative ion-leakage and malondialdehyde content, superoxide anion and hydrogen peroxide showed the opposite situation. Proteomic results showed that a total of 4169 proteins were identified and quantified. Gene Ontology (GO) analysis and Kyoto Encyclopedia of Genes and Genomes (KEGG) analysis indicated that a series of key pathways including carbohydrate metabolism, lipid metabolism, and antioxidant activity were severely damaged by aging treatments. Numerous key proteins such as glyceraldehyde triphosphate glyceraldehyde dehydrogenase, succinate dehydrogenase, lipoxygenase, peroxidase, glutathione-s-transferase and late embryogenesis abundant proteins were significantly down-regulated. However, the up-regulation of the heat shock protein family has made a positive contribution to oxidative stress resistance in seeds. This study provides a useful catalog of the *E. sibiricus* proteomes with insights into the future genetic improvement of seed storability.

## 1. Introduction

Seeds are vital organs for the survival and dispersion of plant species, as well as the fundamental materials for agricultural production and germplasm conservation. However, seeds enter an irreversible and inevitable process of deterioration and aging after natural maturity [1]. With increasing duration of storage, seed vigor gradually decreases until total loss of seed viability. This phenomenon could further result in dramatic loss in economic costs and genetic diversity [2]. Seed aging is the most common form of seed deterioration and is mainly characterized by disturbances in a variety of essential metabolic pathways and cellular biochemical processes including disruption of cellular membranes, mitochondrial dysfunction, and damage to key biological macromolecules such as proteins, lipids, nucleic acids, and carbohydrates [3,4]. Currently, the overproduction of reactive oxygen species (ROS; such as hydrogen peroxide (H_2_O_2_), superoxide anion ($O_{2}^{-}$) etc.) and their direct attack on biological macromolecules is recognized as one of dominant causes of seed aging [5,6,7]. As the by-product of unsaturated lipid peroxidation, malondialdehyde (MDA) is often used as an indicator of measurement of seed aging degree due to excessive accumulation during seed storage [8]. Alternatively, preventing the excessive production of ROS or scavenging the formed ROS were mainly exerted through the antioxidant defense system, which is composed of endogenous antioxidant substances (non-enzymatic system) and antioxidant enzyme systems such as peroxidase (POD) and superoxide dismutase (SOD) [9]. However, it is usually difficult to achieve a complete scavenging ROS due to high seed lipid content and unmanageable ambient storage conditions [3].

In recent years, proteomic techniques based on two-dimensional gel electrophoresis (2-DE) have been extensively employed to elucidate the mechanism of seed aging in some species, such as *Arabidopsis* [10], rice (*Oryza sativa*) [11] and oat (*Avena sativa*) [12]. These investigations declared that many differentially expressed proteins (DEPs) involved in metabolism, energy, protein synthesis, cellular defense and rescue during seed aging. For example, the protein l-isoaspartyl-O-methyltransferase (PIMT) functions to eliminate the excessive accumulation of l-isoaspartic acid and asparagine residues in seed proteins during the aging process, which reduces the sensitivity of seed aging [13]. Furthermore, the down-regulation of storage proteins leads to the inhibition of new protein synthesis and decline of seed vigor, whereas the up-regulation of heat shock proteins (HSPs) improves seed storability [6]. However, just a small number of proteins can be separated and identified by a conventional 2-DE technique because of its limited resolution. Compared to the 2-DE technique, mass spectrometry (MS)-based proteomics is a powerful tool for large-scale protein identification and quantification. Recent technological advances of liquid chromatography coupled to tandem mass spectrometry (LC-MS/MS) have permitted high-throughput detection and quantification of thousands of proteins in biological samples [14]. At present, two popular quantitative proteomic approaches have been developed including label-free quantification (LFQ) and an isobaric labeling strategy such as tandem mass tags (TMT) and isobaric tags for relative and absolute quantitation (iTRAQ) [15,16]. Additionally, the isobaric labeling approach has faster and more stable results than label-free shotgun method [17]. So far, TMT technology has been utilized to explore the molecular mechanism of seed aging in some cereal crops, such as oat [18] and wheat (*Triticum aestivum*) [17].

Siberian wildrye (*Elymus sibiricus*), as the model species of genus *Elymus* belonging to the Triticeae tribe of the Poaceae family, is one of the perennial native grass species with high nutritional value, high yield and resistance to cold and drought, which plays a vital role in forage production and restoring degraded alpine grasslands in the Tibetan Plateau [19,20]. However, the seed vigor of *E. sibiricus* is easily decreased significantly with the extension of storage time under normal local storage conditions [21]. In the eastern Tibetan Plateau, one of the major cultivation regions of Siberian wildrye, seed aging is often exacerbated because of relatively high humidity and frequent rainfalls during the seed harvest season. For instance, the percentage of seed germination at local storage in the fifth year dropped to less than 30% of the initial level (unpublished data). A few previous works about aging of *E. sibiricus* seeds mainly focused on the exploration of artificial accelerated aging conditions [22], improvement of antioxidant enzymes activity by exogenous ascorbic acid (AsA) [23] and reduced genetic integrity [24] of germplasm during seed aging. However, the types and dynamic alteration of possible DEPs involved in the seed deterioration process of *E. sibiricus* has not been extensively described. Here, we utilized the one year-stored *E. sibiricus* seeds, subjected them to artificial aging treatments on different time points, and examined their physiological, biochemical and proteomics status, which could provide basic data and technical theoretical support for the genetic improvement of seed storability and germplasm preservation of *E. sibiricus*.

## 2. Results

### 2.1. Seed Germination Characteristics and Physio-Biochemical Changes of Artificial Aging Treatments (AAT)

**Figure 1:**
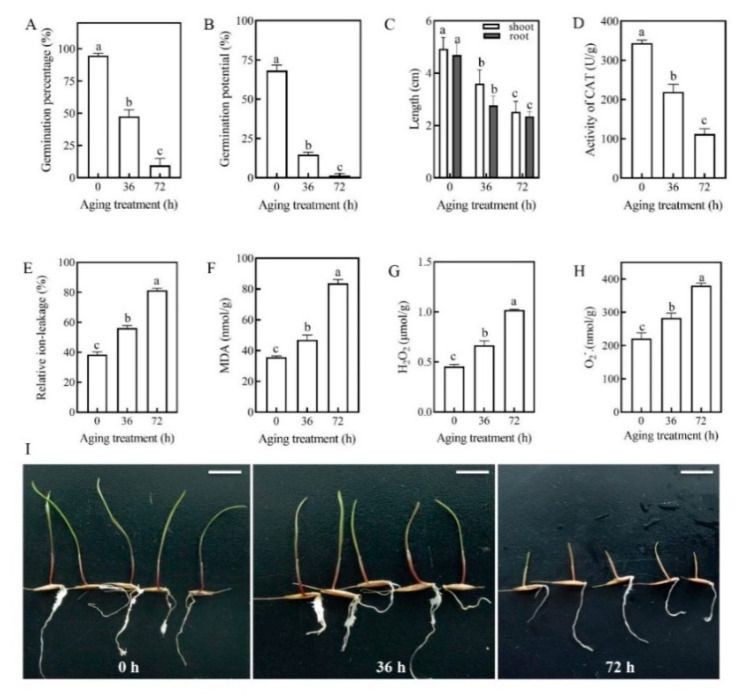
The seed germination characters and physio-biochemical status of *E. sibiricus* seeds with different aging treatments. (**A**) Germination percentage of *E. sibiricus* were calculated every day until 14 days; (**B**) Germination vigor in different aging time; (**C**) comparison of shoot length and root length in response to different aging treatments 14 days after germination; (**D**) the activity of catalase (CAT); (**E**) Relative ion-leakage (RIL); (**F**) malondialdehyde (MDA) content; (**G**) H_2_O_2_ content_;_ (**H**) $O_{2}^{-}$ content; (**I**) Phenotypes of *E. sibiricus* seeds applied by aging treatments (Bar = 1 cm). The different lowercase letters indicate significant differences at *p* < 0.05 and all treatments were conducted with three biological replicates.

To learn more about phenotypic and physiological changes of *E. sibiricus* seed in response to artificial aging treatments (AAT), *E. sibiricus* seed samples with three biological replicates on 0 h, 36 h and 72 h were employed to germination characteristics and physio-biochemical testing (Figure 1). The result implied that the germination percentage of *E. sibiricus* seeds without AAT was dramatically decreased compared with that at 36 h and 72 h of ATT (Figure 1A). Furthermore, the germination potential of the *E. sibiricus* seeds also significantly decreased (Figure 1B). Moreover, the shoot length and root length (Figure 1C,I) also showed a progressive decline (*p* < 0.05). As one of major antioxidants during seed aging, enzymatic activities of CAT (catalase) also showed a significantly decreased trend (*p* < 0.05) along with the increase of the aging time (Figure 1D).

As mentioned above, plant plasma membrane could be affected during artificial aging. To verify the integrity of the plasma membranes, the value of relative ion-leakage (RIL) and the MDA contents in *E*. *sibiricus* seeds with AAT on three time points (0 h, 36 h and 72 h) were measured. The results showed that both of RIL and MDA contents were increased with aging (Figure 1E,F). Furthermore, we also measured the contents of H_2_O_2_ and $O_{2}^{-}$ which are considered the two major ROS in plants. The results indicated that both H_2_O_2_ and $O_{2}^{-}$ overproduced strongly with the extension of artificial aging (Figure 1G,H).

### 2.2. Changes in Protein Abundance after AAT

To obtain a global profile of the quantitative proteome, seeds by three biological replicates on each 0 h, 36 h and 72 h of AAT were employed with TMT labeling and quantified by LC-MS/MS. The result showed that a total of 5134 protein groups were identified at 95% confidence level, among which 4169 proteins were quantified. In order to classify DEPs, three comparison groups (36 h/0 h, 72 h/0 h, and 72 h/36 h) were constructed respectively. A total of 355 proteins (fold change ≥ 1.3 or ≤ 0.77, and *p* < 0.05) were differentially expressed in response to AAT (Table S1).

**Figure 2:**
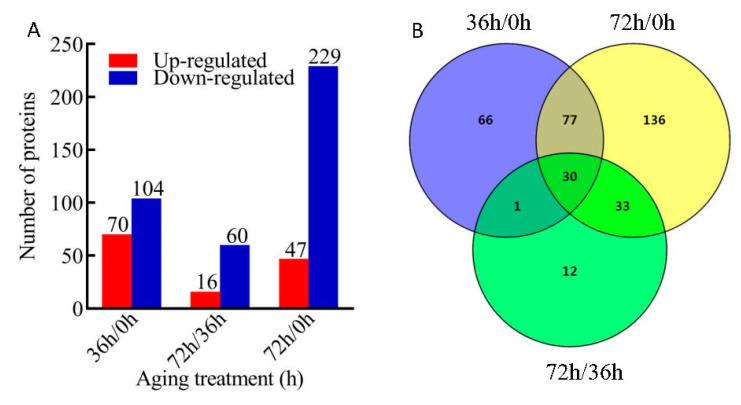
The profile of the differentially expressed proteins (DEPs). (**A**) Number of DEPs identified in different comparison groups with different artificial aging treatments (AAT); (**B**) Venn diagram illustrating the number of identified proteins in the 3 different comparison groups (36 h/0 h, 72 h/0 h and 72 h/36 h). The fold-change cutoff was set when proteins with quantitative ratios over 1.3-fold change was considered up-regulation while quantitative ratio less than 1/1.3-fold change was considered as down-regulation. There are three biological repeats for each treatment.

In detail, a total of 174 DEPs were identified in group 36 h/0 h consisting of 70 proteins up-regulated and 104 proteins down-regulated. In group 72 h/36 h, 16 proteins were up-regulated, and 60 were down-regulated. In group 72 h/0 h, a remarkably bigger number (276) of DEPs were identified with 47 proteins down-regulated and 229 proteins up-regulated (Figure 2A). Interestingly, in the overlapping region of the 3 comparison groups, we found only 30 DEPs including 3 proteins (A0A1D6C821, W5ECA4, W5FD68) were constantly up-regulated and 27 proteins were down-regulated in all of DEPs (Figure 2B). 

### 2.3. Gene Ontology (GO), Kyoto Encyclopedia of Genes and Genomes (KEGG) and Subcellular Location Analyses of Differentially Expressed Proteins (DEPs) in Response to Seed Aging

**Figure 3:**
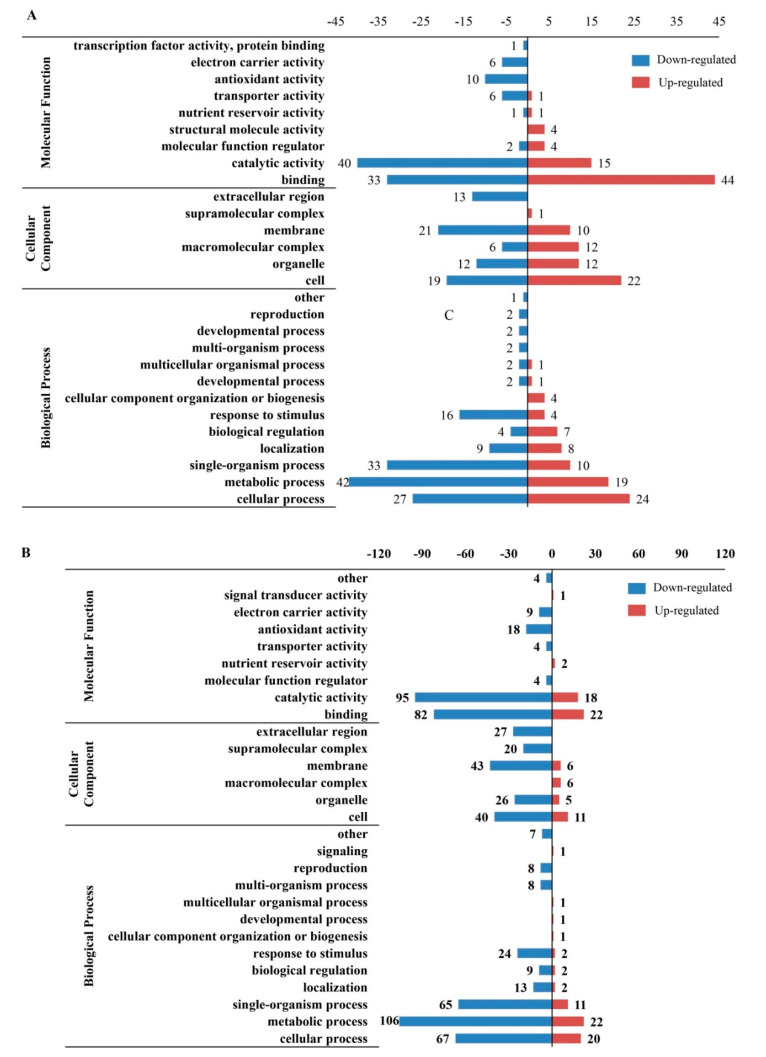

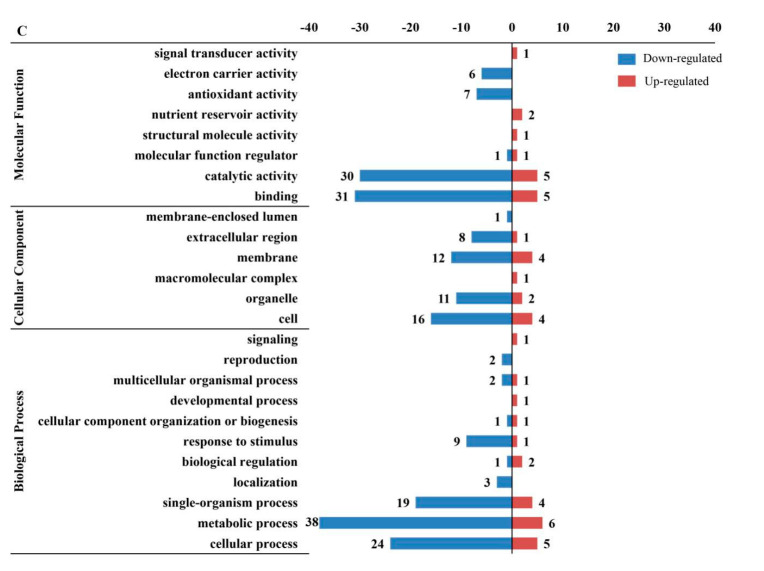
The functional distribution of DEPs in response to AAT by Gene Ontology (GO) level 2. The *x*-axis represents the number of enriched DEPs by GO annotation)and the note number mean protein number. (**A**) the DEPs of 36 h/0 h by GO; (**B**) the DEPs of 72 h/0 h by GO; (**C**) the DEPs of 72 h/36 h by GO.

The result of Gene Ontology (GO) annotation (GO, http://www.ebi.ac.uk/GOA/, 12.19.2019) indicated that all identified DEPs were classified to 14 different biosynthetic processes in which the major categories were response to metabolic process, cellular process, single-organism process, and response to stimulus. It is worth noting that the DEPs involved in the developmental process were only detected in down-regulated DEPs in the comparison group 36 h/0 h (Figure 3A). Among cellular components, cell, membrane, organelle and macromolecular complex were the most abundant groups but the membrane-enclosed lumen was only detected in down-regulated DEPs in 72 h/36 h (Figure 3B). Additionally, in terms of molecular functions, the annotations such as binding, catalytic activity and antioxidant activity were overrepresented in DEPs (Figure 3A–C). Furthermore, the GO annotations such as antioxidant activity, electron carrier activity, transcription factor activity, and protein binding were only enriched in down-regulated DEPs identified in 36 h/0 h and 72 h/0 h comparison groups.

**Figure 4:**
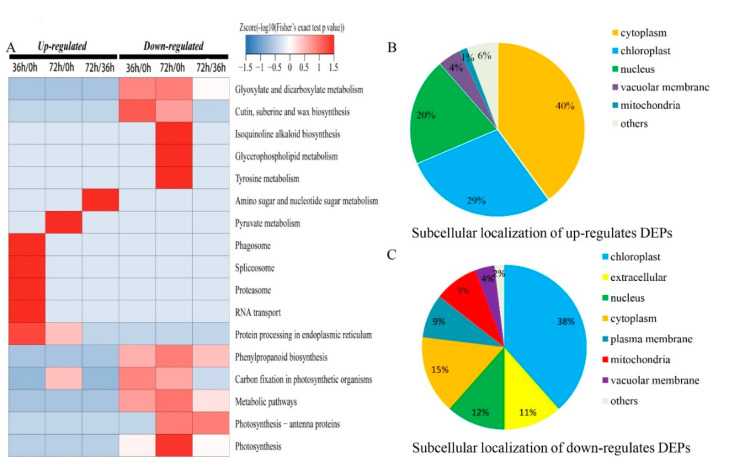
Kyoto Encyclopedia of Genes and Genomes (KEGG) enrichment analysis and subcellular localizations of DEPs. (**A**) the KEGG pathway; (**B**) the subcellular of up-regulated DEPs; (**C**) the subcellular of down-regulated DEPs.

The result of Kyoto Encyclopedia of Genes and Genomes (KEGG) analysis (KEGG, http://www.genome.jp/kegg/, 12.20.2019) showed that most up-regulated DEPs in 36 h/0 h enriched in pathways of phagosome, spliceosome, proteasome, RNA transport and protein processing in endoplasmic reticulum, whereas proteins involved in glyoxylate and dicarboxylate metabolism, phenylpropanoid biosynthesis, metabolic pathways, etc., were down-regulated. Most DEPs related to pyruvate metabolism, protein processing in endoplasmic reticulum, and carbon fixation in photosynthetic organisms increasing, whereas DEPs associated with glycerophospholipid metabolism, tyrosine metabolism, isoquinoline alkaloid biosynthesis decreased in 72 h/0 h. For the 72 h/36 h DEPs, the proteins involved in amino sugar and nucleotide sugar metabolism, metabolic pathways, etc., were up-regulated, whereas proteins in metabolic biosynthesis of phenylpropanoid, photosynthesis-antenna proteins, etc., were down-regulated (Figure 4A). Additionally, the subcellular localizations of the major DEPs were classified to be targeted to cytoplasm, chloroplasts, nucleus, vacuolar membranes and mitochondria (Figure 4B,C).

### 2.4. Key Proteins among DEPs in Response to AAT

A number of important proteins associated with carbohydrate metabolism, lipid metabolism, antioxidant activities and stress response, dramatically changed in response to AAT. In the current study, 61 (44 down-regulated, 19 up-regulated) and 128 (106 down-regulated and 22 up-regulated) metabolic-related proteins in 36 h/0 h and 72 h/0 h were radically changed, respectively (Table S1). The DEPs of metabolic related-protein such as probable UDP-arabinose 4-epimerase 2 and Endo-beta-1,3-glucanase, which were involved in the starch and sucrose metabolism pathways, were both down-regulated remarkably. Furthermore, the down-regulation of the acyl CoA binding protein, like in the tricarboxylic acid cycle (TCA cycle), may cause insufficient energy supply during seed germination (Table 1). Furthermore, we also identified lots of antioxidant and stress response proteins in DEPs such as peroxidase, glutathione-s-transferase (GST), heat shock protein and late embryogenesis abundant protein (LEA), etc., which showed significant abundance alterations (fold change > 1.3, *p* value < 0.05) in response to seed aging (Table 1). 

### 2.5. Candidate Functional Protein Selections and Parallel Reaction Monitoring (PRM) Validation

Among the DEPs in 36 h/0 h and 72 h/0 h, those having predicted annotations related to “metabolism”, “protein binding”, “oxidation-reduction process”, “lipid transport” and “signaling pathways” are most likely to be responsive to AAT. Therefore, we annotated the DEPs that concentrated on the two comparisons i.e., 36 h/0 h and 72 h/0 h groups. In order to validate the TMT results, 20 DEPs were randomly selected for targeted parallel reaction monitoring (PRM) assay, of which 13 proteins had quantitative information and showed a good consistency with TMT results (r^2^ = 0.898, *p* < 0.01), including 10 up-regulated and 3 down-regulated proteins in both 36 h/0 h and 72 h/0 h groups (Table 2)

## 3. Discussion

### 3.1. Changes of Carbohydrate Metabolism-Related DEPs during AAT

During long term storage of seeds, proteins related to nutrition storage, energy supply, and stress responses can be dramatically changed [1]. This change might in turn lead to a significant decrease in the germination rate, germination potential as well as shoot length and root length of artificially aged seeds (Figure 1A–C). [1] This study indicated that lots of down-regulated proteins in carbohydrate metabolism were mainly enriched in pathways of starch and sucrose metabolism, glycolysis and the TCA cycle, which led to impaired seed metabolism and energy supply, and eventually decreased seed germination and vigor.

It has been found that the seed vigor loss among wheat seed samples with different aging treatment was related to a significant reduction of sucrose (SU) and a slight enhancement of raffinose (Ra) as well as a subsequent increase in Ra/SU ratio [25]. In this study, we identified several down-regulated DEPs involving starch and sucrose metabolism pathways including UDP-arabinose 4-epimerase 2 (W5B7S6), endo-beta-1,3-glucanase (Q1ERG1), glucan endo-1,3-beta-glucosidase 3-like isoform X1 (A0A1D5UYF9), fructose-bisphosphate aldolase (W5EDL3), beta-glucosidase BoGH3B-like (A0A1D5Z7K1), beta-glucosidase 31-like (A0A1D6S663), glucan endo-1,3-beta-glucosidase 4-like isoform X2 (A0A1D5ZSY5), serine/threonine-protein kinase (A0A1D5TQZ5) and so on (Table 1). The down-regulation of these key proteins in starch and sucrose metabolism pathways might cause impairment of the energy supply chain during seed aging.

Glycolysis is the major biochemical process of the carbohydrate metabolism responsible for the conversion of glucose or glycogen to pyruvate or lactic acid in the cytoplasm, which produces a small amount of ATPs under anaerobic or hypoxic conditions [26]. Here, we observed a significant decrease in several isoforms of the glyceraldehyde triphosphate dehydrogenase (GAPDH, A0A1D5TTT1, A0A1D5YL69) of glycolysis at 36 h and 72 h compared to 0 h (Table 1). As is well known, the GAPDH transforms oxidatively phosphorylate glyceraldehyde 3-phosphate (G3P) into 1,3-bisphosphoglycerate (1,3-BPG) in the glycolysis pathway. The downregulation of GAPDH indicates an overall down-regulation of glycolysis during seed aging, and it could also be considered as an important indicator in the seed aging process of Siberian wildrye.

Moreover, numerous studies have shown that up-regulated proteins in the TCA cycle can provide energy for seeds germination [27]. In this study, lots of TCA cycle-related proteins including succinate dehydrogenase (SDH) subunit 6 (A0A1D6CTY7) in mitochondria, acyl-CoA-binding protein-like (A0A1D6D5I2) were significantly reduced, and phosphoenolpyruvate carboxylase 2 (W5FCI5) in the cytoplasm was up-regulated at 36 h and 72 h compared to 0 h (Table 1). It is reported that SDH, a marker enzyme for mitochondria, decreased in both cotyledons and embryonic axis tissues of aged sunflower seeds and leads to delayed growth of seedlings [28]. Furthermore, the SDH1 mutant reduced the activity of the electron transport chain in mitochondrial, which may cause rapid overproduction of ROS during seed germination and response to oxidative damage in Arabidopsis [29]. In consequence, down-regulated SDH enzyme during artificial aging could break redox balance between oxidative and reductive substances by ROS overproduction and then disrupted the respiratory capacity of mitochondrial, eventually resulting in poor germination vigor of aged seeds. 

### 3.2. Changes of Lipid Metabolism-Related Proteins during AAT

Lipoxygenase (LOX) is an essential enzyme for fatty acid oxidation in plants. It not only catalyzes the production of fatty acid derivatives and ROS by phenolic glycerides, but is also strongly related to plant disease resistance and anti-injury responses [30,31]. In this study, the lipoxygenase (A0A1D6CGK6), non-specific lipid transfer protein GPI-anchored 1 (A0A1D6CX16) and diacylglycerol kinase (A0A1D6B2R1) abundance decreased continuously at 36 h and 72 h compared to 0 h. This indicated that seed aging could inhibit the process of enzymatic-lipid oxidation and reduced the defense against the harsh environment. However, the physio-biochemical measurement results indicated that both RIL and MDA increased with aging time, implying the plasma membrane was significantly destroyed (Figure 1E,F). This result also suggests that the overproduction of MDA, the final product of lipid peroxidation, was possibly produced by lipid peroxidation through non-enzymatic pathways instead of the enzymatic pathways [25,31].

### 3.3. Antioxidant Activities Responded to AAT

Generally, seeds under long-term storage are liable to suffer oxidative damage owing to the accumulation of ROS as well as the decrease of the antioxidant capacity in cells which accelerates the disturbances of the cellular redox homeostasis and a loss of germination ability [32]. The “redox homeostasis” is regulated by antioxidant activities species including the enzymatic (POD, SOD, CAT, etc.) and the non-enzymatic antioxidative systems such as glutathione (GSH), glutathione reductase (GR), and other antioxidants [33]. 

Broadly, peroxidases (PODs) are often heme-containing antioxidant enzymes, and they are divided into three superfamilies based on their structural and catalytic properties [34,35]. In particular, the three peroxidase superfamilies from plant source mainly consist of catalases (CAT), ascorbate peroxidase (APx), peroxidase (POX, EC 1.11.17, special indications of plant peroxidase) and glutathione peroxidase (GPx), which efficiently protect plants against damage by removing ROS in various biological processes [34]. Several studies have shown that the significant decrease activity of peroxidases is one of the landmark events of seed aging in rice [33], oat [36], and other crops. In the present study, we identified several down-regulated peroxidases such as A0A1D5V3N8, A0A1D5WDA8, A0A1D6CQN7, A0A1D6CV94, W4ZYX8, W5FEC7 and W5G6B5 (Table 1). Furthermore, we also found that the activity of CAT decreased dramatically in response to AAT (Figure 1D). Antioxidant enzymes such as CAT and POX are responsive to stresses when scavenging extra ROS to maintain the intracellular oxidative balance [37]. However, ROS overproduction increases lipid peroxidation and inhibits activities of antioxidant enzymes, triggering the inability of the cells to completely scavenge the radicals [38]. This may be part of the reason why the content of H_2_O_2_ and $O_{2}^{-}$ had a positive trend with CAT activities over aging time of *E. sibiricus* (Figure 1D,G,H).Additionally, glutathione-s-transferase (GST) is a key enzyme that catalyzes the reaction of GSH and substrates and takes an essential role in primary and secondary metabolisms, stress metabolism, detoxification as well as seedling development [39,40]. It is worth noting that the overexpression of the GST gene such as *PpGST* in *Pyrus pyrifolia* and *GmGSTU4* in soybean significantly scavenged excess ROS and improved plant resistance to drought and salt [41]. Moreover, Cheng et al. [42] indicated that the dysfunction of GST, a member of the ascorbate-glutathione (AsA-GSH) antioxidant system, is attributed to the main cause of loss of seed vigor in artificially aged seed of oats. In this study, we found that the probable glutathione-s-transferase BZ2 (A0A1D6CAM6) showed a significant decrease at 36 h and 72 h compared to 0 h post AAT (Table 1). This result indicated that the reduction of GST may also cause excessive accumulation of ROS under oxidative stress, eventually resulting in decreased seed vigor and even seed death.

### 3.4. The Role of Stress-Related Proteins during AAT

LEA proteins are a group of key hydrophilic proteins during plant growth and seed development, which have been related to plant response to stresses including protecting cellular structures and improving cell thermal stability, and serving as a molecular chaperone protein to resist cell damages [43]. Wang et al. indicated that the overexpression of *OsLEA* enhanced the resistance to drought stress at seedling stage of rice [44]. Hundertmark et al. confirmed that the decrease of LEA proteins degraded the seed longevity in *Arabidopsis* seeds [45]. In this case, LEA protein (A0A024CKY0) and an isoform of 11 kDa LEA protein-like (A0A1D5ZWT3) were down-regulated at 36 h and 72 h compared to 0 h post AAT, implying that these LEAs might be associated with decreased seed vigor and germination in aged seeds of *E. sibiricus*. 

Heat shock proteins (HSP), mainly located in the cytoplasm under normal physiological conditions, are widely distributed in animals and plants, including HSP 90, HSP 101, HSP 70, etc. [46]. When plants are subjected to high temperature stress, HSP is significantly up-regulated to reduce the excessive oxidative stress and maintain the correct spatial conformation of protein [47]. It is worth noting that the HSP 90 could regulate temperature-dependent seedling growth through stabilizing the auxin co-receptor F-box protein TIR1 in *Arabidopsis* [48]. Furthermore, the overexpression of HSP 101 improved the resistance to high temperatures in transgenic *Arabidopsis* seedlings [49]. In this study, the up-regulated HSP 90 (F4Y590), HSP 101 (Q9SPH4), chaperone protein ClpB1-like isoform X1 (W5FD68) and heat shock 70 kDa protein 14-like (A0A1D6DGU9) were observed at 36 h and 72 h compared to 0 h post AAT respectively (Table 1). These results demonstrated that the HSPs could improve the resistance of Siberian wildrye seeds under high temperature and humidity conditions, as confirmed by some similar investigations in *Arabidopsis* [47], oat [12] and other species [50].

Ribulose-1,5-bisphosphate carboxylase/oxygenase (RuBisCO), which localized to the stroma of chloroplast, is a principal enzyme for CO_2_ fixation reaction in the Calvin cycle of plant photosynthesis [51]. Overexpression of RuBisCO increased the biomass productivity and the resistance to high temperature in seedlings of microalgae (*Nannochloropsis* spp.) and *Arabidopsis* [52]. However, when plants are subjected to stresses such as high temperature or tropospheric ozone (O_3_) etc., the activities or content of RuBisCO could be significantly decreased [53]. Here, down-regulation of the RuBisCO small chain (A0A1D5Z4N3) was observed at 36 h and 72 h compared to 0 h post AAT (Table 1), implying AAT could restrain the RuBisCO activity and reduce the plant biomass and shoot length and root length (Figure 1C,I). 

## 4. Materials and Methods 

### 4.1. Plant Materials

The seeds of Siberian wildrye (*Elymus sibiricus* L. cv. ‘Chuancao No.2′) were harvested in Hongyuan county, Aba prefecture, Sichuan province of China in September 2018 (102.5441 E, 32.7734 W, 3500 m), which is located in the southeast of Qinghai-Tibetan Plateau. The original germination rate and moisture content was 95.01% ± 0.32 and 8.31% ± 0.25, respectively. The seeds with similar size and weight were selected and then stored at −20 °C.

### 4.2. Artificial Aging Treatment and Germination Test

AAT and the germination test were employed with the method described in the previous research with little modification [12]. Briefly, 150 g seeds which were packed in an aging chamber (LH-160, Zhejiang TOP Yunnong Technology Co., Ltd., Hangzhou, China) maintained at 45 ± 1 °C and 90 ± 1% relative humidity (RH), for 0 h, 36 h and 72 h. Each treatment had three replications containing 100 seeds, which were moistened to saturation with distilled water. Then the seeds were cultured in a germination chamber (GXZ, Ningbo, China) with a temperature of 25 °C and 70% relative humidity with 8/16 h light/darkness. Seed germination rate was calculated every day until 14 days and 10 seedlings of each replicate were randomly selected to measure the shoot length and root length after germinating 14 days. The calculation criterion of all the indexes was as follows. The germination rate was the proportion of seedlings to the number of seeds. The germination vigor was calculated based on the proportion of seedlings in the seventh day to the total number of seeds. Finally, the 0 h, 36 h and 72 h aged seeds, were stored at −80 °C for further analysis. All samples were three biological repeats. 

### 4.3. Monitoring the Relative Ion-Leakage and Malondialdehyde Content

The value of RIL was measured as described previously [6]. The relative ion-leakage (%) = (A2 − A1)/(A3 − A1) × 100%. In the formula, A1 represented the initial conductivity. A2 was detected after the seed was shaken for 1 day at indoor temperature (DDS-307, Shanghai, China). A3 was the absolute conductivity of the seed after boiling in a water bath for 1 h. 

The MDA content was measured using a TBA (thiobarbituric acid) method. Briefly, the seeds (0.1 g) were ground, homogenized in 10 mL 10% trichloroacetic acid (TCA), and centrifuged at 1500× *g* for 10 min. Then 2 mL supernatant was isovolumetric mixed with 0.6% TBA and boiled at 100 °C for 10 min. The absorbance values at 532 nm and 600 nm were used to calculate the MDA content. Each sample had three biological repeats.

### 4.4. Measurement of Catalase (CAT) Activity and Reactive Oxygen Species (ROS) Content 

The activities of catalase (CAT) were examined using CAT-2-Y (Solarbio, Beijing, China). Briefly, the seeds were grinded on ice, mixed with 50 mmol PBS buffer (pH = 7.8), and then centrifuged at 2500× *g* for 10 min. Finally, the absorbance value obtained from supernatants were used to calculate the activity of CAT. 

The measurement of $O_{2}^{-}$ and H_2_O_2_ content was based on Gong’s method [54]. Briefly, the $O_{2}^{-}$ reacts with MSDS (hydroxylamine hydrochloride) to generate NO^2−^. Then a red azo compound is generated with the reaction of p-aminobenzenesulfonic acid and α-naphthylamine, which possesses an absorption peak at 530 nm. The yellow titanium peroxide composite generated by the reaction of H_2_O_2_ which has an absorption peak at 415 nm is used to calculate the H_2_O_2_ content. Each sample has three biological repeats. 

### 4.5. Protein Extraction and Trypsin Digestion 

The protein extraction and trypsin digestion were carried out as previous studies with appropriate modifications [17]. Briefly, aged seeds at 0 h, 36 h and 72 h were ground by liquid nitrogen and sonicated for three times on ice using a high intensity ultrasonic processor (Scientz, Zhejiang, China) in lysis buffer (8 mol/L urea, 2 mmol/L ethylenediaminetetraacetic acid (EDTA), 10 mmol/L dithiothreitol (DTT) and 1% Protease Inhibitor Cocktail), and then centrifuged at 20,000× *g* at 4 °C for 10 min. Finally, the protein was reacted with pre-cooled 20% TCA for 2 h at 4 °C. After being cleaned completely with cold acetone, the protein was dissolved with 100 mmol/L TEAB buffer (pH 8.0). Then, the concentration of protein was determined with a 2-D Quant kit. For protein digestion, the protein solution was digested with 5 mmol/L DTT for 30 min at 56 °C and 11 mmol/L iodoacetamide (IAA) for 15 min. Finally, the diluted protein sample was digestion by 1:50 trypsin-to-protein mass ratio for the first digestion overnight and 1:100 trypsin-to-protein mass ratio for a second 4 h-digestion. Each sample has three biological replicates.

### 4.6. Tandem Mass Tags (TMT) Labeling, High-Performance Mass Chromatography (HPLC) Fractionation and Liquid Chromatography-Tandem Mass Spectrometry (LC-MS/MS) Analysis

Peptide reconstitution was performed in 0.5 mol/L TEAB using a 10-plex TMT kit (Thermo, Waltham, MA, USA). Follow by fractionated into fractions by high pH reverse-phase high-performance mass chromatography (HPLC) using Agilent 300Extend C18 column (5 μmol particles, 4.6 mm ID, 250 mm length). Finally, the peptides were dissolved in 0.1% formic acid (FA) for LC-MS/MS analysis by loading onto a constant flow rate of 700 nL/min on an EASY-nLC 1000 ultra-performance liquid chromatography (UPLC) system of reversed-phase analytical column (Thermo, Waltham, MA, USA). 

For the LC-MS/MS analysis, the peptides were subjected to NSI source followed by tandem mass spectrometry (MS/MS) in Orbitrap Fusion TM (Thermo) coupled online to the UPLC. Peptides were selected for MS/MS using the normalized collision energy (NCE) setting as 35 while intact peptides and ion fragments were detected in the Orbitrap at a resolution of 60,000 and 15,000, respectively. A data-dependent procedure that alternated between one MS scan followed by 20 MS/MS scans was applied for the top 20 precursor ions above a threshold intensity greater than 5E3 in the MS survey scan with 30.0 s dynamic exclusion. The electrospray voltage applied was 2.0 kV. Automatic gain control (AGC) was used to prevent overfilling of the orbitrap; 5E4 ions were accumulated for generation of MS/MS spectra. For MS scans, the m/z scan range was 350 to 1550. Fixed first mass was set as 100 m/z.

### 4.7. MS/MS Database Search and Bioinformatics Analysis 

The resulting MS/MS data were processed using MaxQuant with integrated Andromeda search engine (v.1.5.2.8). Tandem mass spectra were searched against Wheat (*Triticum aestivum*) database (www.uniprot.org/taxonomy/4565) concatenated with reverse decoy database. Trypsin/P was specified as cleavage enzyme allowing up to 2 missing cleavages. Mass error was set to 10 ppm (parts per million) for precursor ions and 0.02 Da for fragment ions. The thresholds of unique peptide were determined by false discovery rate (FDR) < 0.01. All the other parameters in MaxQuant were set to default values. For protein quantification, the protein ratios are calculated as the median of only unique peptides of the protein.

Proteins was considered as a DEP if its fold change was >1.3 (up-regulated protein) or <0.77 (down-regulated protein) and its *p* value < 0.05 (Student’s *t*-test). The mass spectrometry proteomics data have been deposited to the ProteomeXchange Consortium via the Proteomics Identification Database (PRIDE) partner repository with the dataset identifier PXD021552.

For the bioinformatics analysis, the DEPs of GO annotation were derived from the UniProt-GOA database. Then DEPs were classified by Gene Ontology annotation based on three categories: biological process, cellular component and molecular function. For the KEGG pathway annotation, we first used the KEGG online service tool KEGG Automatic Annotation Server (KAAS) to annotate protein’s KEGG database description. Then we mapped the annotation result on the KEGG pathway database using the KEGG online service tool KEGG mapper. Subcellular localization was conducted by wolfpsort (wolfpsort, http://wolfpsort.seq.cbrc.jp/, 12.20.2019) predication soft. Cluster membership was visualized by a heat map using the “heatmap.2” function from the “gplots” R-package. 

### 4.8. Parallel Reaction Monitoring Validations

To verify the protein expression levels obtained by TMT analysis, 20 differentially abundant proteins (unique peptides≥ 2, fold change > 1.3 or < 0.77) were randomly chosen based on the TMT results and further quantified by the PRM assay according to Xu’s method [55] at Jingjie PTM-Biolab Co., Ltd. (Hangzhou, China). 

Briefly, peptides were prepared as described above for the TMT assay. These obtained peptide mixtures were subjected to Nitrogen soluble index (NSI) source followed by tandem mass spectrometry (MS/MS) in Q ExactiveTM Plus (Thermo) coupled online to the UPLC. A full MS was performed in the Orbitrap at a resolution of 70,000 (AGC target at 3E6; the maximum injection time at 50 ms and the m/z range was 350–1200), followed by 20 MS/MS scans on the Orbitrap at a resolution of 17,500 (AGC target was 1E5, and the maximum injection time was 100 ms). Mass window for precursor ion selection was 1.6 m/z. The isolation window for MS/MS was set at 2.0 m/z. The NCE was 27% with high energy collision dissociation (HCD). The FDR was set to 0.01 for the proteins and peptides. The resulting MS data were processed using Skyline (v.3.6) program as described before [56]. An analysis of three biological replicates was performed for each sample (0 h, 36 h and 72 h groups, respectively). 

### 4.9. Statistical Analysis

SPSS19.0 software was used to test the variance of vitality parameters, and physiological and chemical indicators between different treatments. All results are shown as the mean ± standard deviation (SD) and the least significant difference (LSD) of the mean by the Duncan test (*p* < 0.05).

## 5. Conclusions

**Figure 5:**
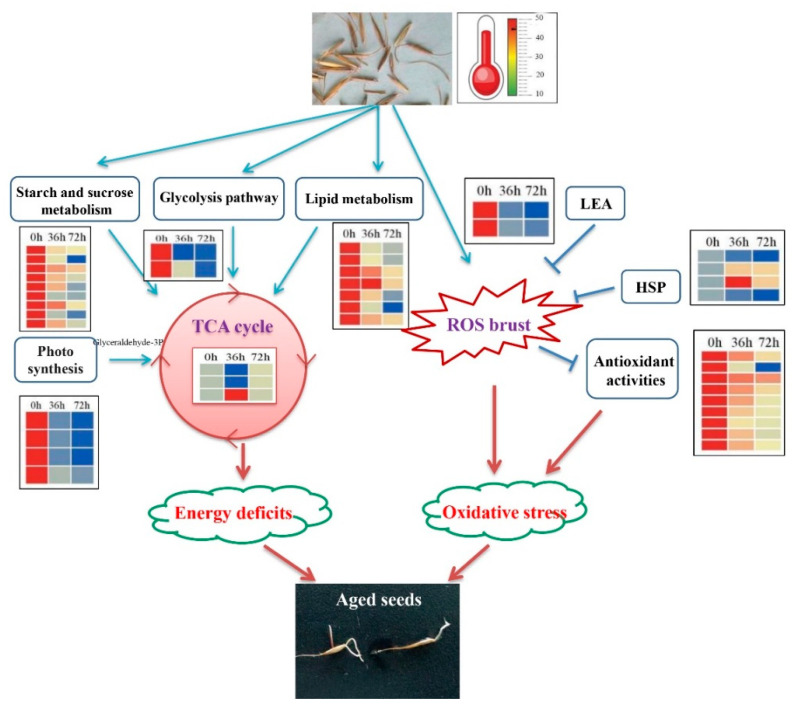
The overview of differentially modulated proteins during AAT. Reactive oxygen species, ROS; Late embryogenesis abundant proteins, LEA; Heat shock proteins, HSP; tricarboxylic acid cycle, TCA cycle.

In summary, a model of AAT-mediated stress response in *E. sibiricus* was proposed (Figure 5). AAT induces ROS accumulation, osmotic pressure, and cell damage in *E. sibiricus* seeds, which significantly reduces the germination rate and seed vigor, and severely inhibits the embryos’ development and seedling growth. Meanwhile, the proteomic analysis revealed changes in a variety of key proteins involved in carbohydrate metabolism, lipid metabolism, antioxidative systems and stress response induced by AAT, which in turn caused energy deficits and imbalance between ROS and the antioxidative defense system. These results provide basic information for exploring the molecular mechanisms that influence the seed aging of *E. sibiricus*. Nevertheless, a challenge for future work will be to elucidate the complex regulation network among those identified DEPs and their function in the context of artificial aging and natural aging.

## Figures and Tables

**Table 1:** Fold changes of DEPs in key pathway in different groups.

Pathway	Accession No.	Protein Name	Log_2_ (Fold Change)		
			36 h/0 h	72 h/0 h	72 h/36 h
glycolysis pathway	A0A1D5TTT1	Glyceraldehyde-3-phosphate dehydrogenase	−1.154c	−1.109	0.045
	A0A1D5YL69	Glyceraldehyde-3-phosphate dehydrogenase	−0.545	−1.092	−0.546
tricarboxylic acid cycle (TCA)	A0A1D6CTY7	Succinate dehydrogenase subunit 6	−0.369	−0.444	−0.075
	A0A1D6D5I2	Acyl-CoA-binding protein-like	−0.379	−0.307	0.072
	W5FCI5	Phosphoenolpyruvate carboxylase 2	0.516	0.561	0.045
starch and sucrose metabolism	W5B7S6	Probable UDP-arabinose 4-epimerase 2	−0.431	−0.484	−0.052
	Q1ERG1	Endo-beta-1,3-glucanase	−0.528	−1.234	−0.706
	A0A1D5UYF9	Glucan endo-1,3-beta-glucosidase 3-like isoform X1	−0.25	−0.387	−0.136
	W5EDL3	Fructose-bisphosphate aldolase	−0.345	−0.576	−0.23
	A0A1D5Z7K1	Predicted: beta-glucosidase BoGH3B-like	−0.318	−0.802	−0.484
	A0A1D6S663	Beta-glucosidase 31-like	−0.667	−0.76	−0.093
	A0A1D5ZSY5	Glucan endo-1,3-beta-glucosidase 4-like isoform X2	−0.165	−0.465	−0.299
	A0A1D5TQZ5	Serine/threonine-protein kinase	−0.666	−1.005	−0.339
	A0A1D6DGU9	Glucan endo-1,3-beta-glucosidase GII-like	−0.3	−0.589	−0.289
lipid metabolism	A0A1D6CGK6	Lipoxygenase	−0.536	−0.664	−0.128
	A0A1D6CX16	Non-specific lipid transfer protein GPI-anchored 1	−0.49	−0.697	−0.207
	A0A077RZ37	Glycerophosphodiester phosphodiesterase GDPD6-like	−0.175	−0.424	−0.248
	A0A0A0S1W1	12-oxo-phytodienoic acid reductase 2	0.01	−0.396	−0.405
	A0A1B5GFV1	Caleosin	−0.378	−0.828	−0.45
	A0A1B5GFV6	Caleosin	−0.548	−1.207	−0.659
	A0A1D6B2R1	Diacylglycerol kinase	−0.294	−0.406	−0.113
antioxidant activities	A0A1D5V3N8	Peroxidase	−0.353	−0.743	−0.391
	A0A1D5WDA8	Peroxidase	−0.946	−3.095	−2.148
	A0A1D6CQN7	Peroxidase	−0.269	−0.387	−0.118
	A0A1D6CV94	Peroxidase	−0.396	−0.717	−0.321
	W4ZYX8	Peroxidase	−0.448	−0.901	−0.453
	A0A1D5WT84	Peroxidase	−0.623	−0.896	−0.273
	W5FEC7	Peroxidase	−0.783	−0.923	−0.14
	W5G6B5	Peroxidase	−0.575	−0.997	−0.422
	A0A1D6CAM6	Probable glutathione S-transferase BZ2	−0.507	−0.855	−0.348
anti-stress protein	A0A024CKY0	LEA protein	−0.555	−0.683	−0.128
	A0A1D5ZWT3	11 kDa LEA protein-like	−0.487	−0.561	−0.073
	F4Y590	Heat shock protein 90	0.51	0.288	−0.222
	Q9SPH4	Heat shock protein 101	0.448	0.418	−0.03
	W5FD68	Chaperone protein ClpB1-like isoform X1	0.89	0.436	−0.454
	A0A1D6DGU9	Heat shock 70 kDa protein 14-like	0.407	0.155	−0.252
	A0A1D5Z4N3	Ribulose bisphosphate carboxylase small chain	−1.115	−1.428	−0.313

Note: Fold change > 1.3-fold or cut off less than 0.77-fold were considered up-regulated or down-regulated (*p* value < 0.05), respectively. Fold changes in Table 1 were carried out log_2_ (fold change).

**Table 2:** Comparison between the isobaric tandem mass tag (TMT) labeling for relative quantitation and parallel reaction monitoring (PRM).

Protein Accession	Protein Description	36 h/0 h Ratio (TMT)	36 h/0 h Ratio (PRM)	72 h/0 h Ratio (TMT)	72 h/0 h Ratio (PRM)
A0A1D6B2D8	Carboxypeptidase	0.84	0.82	0.73	0.67
W5AC28	Glutathione-S-transferase DHAR2-like	0.69	0.64	0.74	0.81
Q8GVD3	Thioredoxin	0.74	0.67	0.79	0.72
A0A1D6RL87	26S proteasome non-ATPase Regulatory subunit 1 homolog A-like isoform X4	1.37	1.50	1.24	1.46
Q2PCD2	Non-specific lipid-transfer protein	0.81	0.87	0.75	0.72
A0A1D5WTN1	Predicted: transmembrane protein 214-B	0.89	0.77	0.76	0.73
A0A1D5Z9W3	Elongation factor 2-like	0.84	1.34	0.73	1.31
P11383	Ribulose bisphosphate carboxylase large chain	0.34	0.16	0.21	0.09
P12112	Adenosine triphosphate (ATP) synthase subunit alpha, chloroplastic	0.93	1.08	0.76	0.84
F4Y590	Heat shock protein 90	1.42	1.56	1.22	1.40
A0A1D5ZWT3	11 kDa late embryogenesis abundant protein-like	0.71	0.72	0.68	0.71
A0A1D6C2V8	Transmembrane protein 120 homolog	0.86	0.77	0.75	0.63
W5D591	Small ubiquitin-related modifier	0.79	0.79	0.67	0.65

## Supplementary Materials

The following are available online at https://www.mdpi.com/2223-7747/9/10/1370/s1, Table S1: The DEPs of three groups of Siberian wildrye.

## Abbreviations

ABA	abscisic acid
CAT	catalase
GA3	gibberellin
GAPDH	glyceraldehyde triphosphate glyceraldehyde dehydrogenase
GO	Gene Ontology
GST	glutathione-S-transferase
H_2_O_2_	hydrogen peroxide
Hsps	heat shock proteins
KEGG	Kyoto Encyclopedia of Genes and Genomes
LC-MS/MS	liquid chromatography-tandem mass spectrometry
LEAs	late embryogenesis abundant proteins
LOX	lipoxygenase
MDA	malondialdehyde
$O_{2}^{-}$	superoxide anion
-OH	hydroxyl radicals
PRM	parallel reaction monitoring
RIL	relative ion-leakage
ROS	reactive oxygen species
RuBisCO	Ribulose-1,5-bisphosphate carboxylase/oxygenase
SDH	succinate dehydrogenase
SOD	superoxide dismutase
TMT	tandem mass tags
